# A CARE-compliant article: A case report of scoliosis complicated with multicentric carpotarsal osteolysis

**DOI:** 10.1097/MD.0000000000017828

**Published:** 2019-11-27

**Authors:** Kunihiko Miyazaki, Satoshi Komatsubara, Koki Uno, Ryuji Fujihara, Tetsuji Yamamoto

**Affiliations:** a Department of Orthopaedic Surgery, Faculty of Medicine, Kagawa University, Kita-gun, Kagawa; b Department of Orthopaedic Surgery, National Hospital Organization Kobe Medical Center, Hyogo, Japan.

**Keywords:** multicentric carpotarsal osteolysis, osteolysis, scoliosis, spine, treatment

## Abstract

**Rationale:**

Multicentric carpotarsal osteolysis (MCTO) is a rare hereditary disease caused by mutations in *MafB*, a negative regulator of osteoclastogenesis.

**Patient concerns:**

A 20-year-old, Japanese woman with scoliosis visited our institute for treatment. Scoliosis was apparent since she was 12 years old, but she had not sought treatment until the age of 19. Medical examination showed a typical facial appearance associated with a small forehead and hypotelorism; shortening of the fingers of both hands and both upper limbs was observed, in addition to clubfoot. No café au lait spots or mental retardation were observed. On the other hand, the trunk showed evidence of an irregular waistline and a rib hump that obviously suggested scoliosis. Neurological deficit was not observed. Spirometry showed decreased forced vital capacity (FVC). Although proteinuria was observed, renal dysfunction and hypertension were not seen. The major curve of scoliosis was 82° (MC, Th7–L2; Th11 apical vertebra), and the upper curve was 77° (UC, Th1-6; Th3 apical vertebra). In a recumbent-traction position, the major curve was 54° and the upper curve was 56°. The pelvic incidence minus lumbar lordosis (PI–LL) angle was <10° and no mismatch was observed; thoracic kyphosis was decreased to 16°.

**Diagnosis:**

The patient was diagnosed with symptomatic scoliosis secondary to MCTO.

**Interventions:**

We decided to perform a correction and fusion from Th2 to L3 using a posterior spinal instrumentation.

**Outcomes:**

Postoperative x-ray demonstrated scoliosis angle correction from 77° to 38° at Th1–6 and 82° to 39° at Th7–L2. Postoperative x-ray demonstrated thoracic kyphosis angle correction from 16° to 21°. The patient's height increased from 155 to 161 cm.

**Lessons:**

It has been 24 months since the operation, and no exacerbation has been observed. To the best of our knowledge, this is the first report of surgical treatment of scoliosis secondary to MCTO.

## 1 Introduction

Multicentric carpotarsal osteolysis (MCTO) is a rare hereditary disease caused due to mutations in the *MafB* gene, a negative regulator of osteoclastogenesis.

In most cases of MCTO, osteolysis develops mainly in the patient's carpal bones and tarsus from infancy, and chronic renal failure often arises as a complication. MCTO is also known as idiopathic multicentric osteolysis with nephropathy.^[1]^

Hardegger et al^[2]^ have classified idiopathic multicentric osteolysis into 5 types; type 1, hereditary multicentric osteolysis with dominant transmission; type 2, hereditary multicentric osteolysis with recessive transmission; type 3, idiopathic multicentric osteolysis with nephropathy; type 4, Gorham massive osteolysis; and type 5, Winchester syndrome. MCTO is classified as type 3. Lagier and Rutishauer^[3]^ reported cases of type 3 osteolysis complicated with kyphosis or scoliosis. However, the number of such cases is small considering the total number of osteolysis cases.^[3–6]^ A surgical case of scoliosis secondary to Gorham massive osteolysis, classified as type 4, has been reported previously.^[7]^ We present a case of scoliosis complicated with MCTO classified as type 3 osteolysis. To our knowledge, this is the first report describing surgical treatment of scoliosis secondary to MCTO. Patient has provided informed consent for publication of the case.

## 2 Patient information/clinical findings

**Figure 1:**
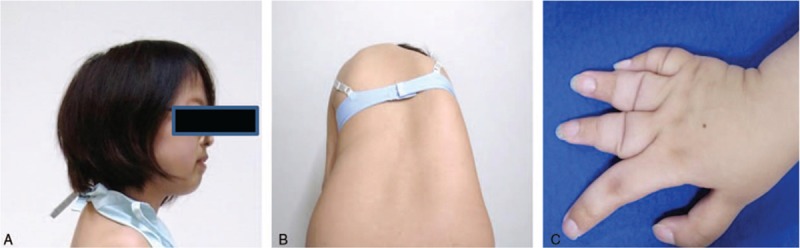
(A) Small chin; (B) rib hump; (C) shortening of fingers of the hand.

A 20-year-old Japanese woman visited our institute for treating her scoliosis. She was a second-born child among dichorionic diamniotic twins. She had visited a pediatrics division at another hospital because she experienced restriction in the range of the motion in both elbow joints, and clubfoot. When the patient was 3 years old, she was diagnosed with focal segmental glomerulosclerosis due to proteinuria. At the age of 7 years, she was diagnosed with MCTO based on multiple osteolysis and a typical facial appearance associated with a small forehead and hypotelorism. At the age of 18 years, she underwent genetic testing; a *MafB* missense polymorphic mutation was identified, and a definitive diagnosis was established. Scoliosis was apparent since she was 12 years old, but she had not sought treatment until the age of 19. The patient weighed 38.6 kg and had a height of 155 cm. Medical examination showed a typical facial appearance associated with a small forehead and hypotelorism; shortening of the fingers of both hands fingers and both upper limbs were observed in addition to clubfoot. No café au lait spots or mental retardation were seen. On the other hand, the trunk showed evidence of an irregular waistline and a rib hump that obviously suggested scoliosis (Fig. 1).

Due to the remarkable deformation of upper limbs beyond the elbow joint, upper limb muscular strength could not be evaluated accurately. Further, due to the remarkable deformation of lower limbs beyond the ankle joint, lower limb muscular strength could not be evaluated accurately. Other muscle weakness was not present. There were no walking problems. No hypoesthesia, numbness, or dysuria was found. Hyperreflexia was observed in the upper and lower limbs, but no neurological deficit was observed. No laterality was found in the limbs and Babinski reflex was positive. Spirometry showed a decrease in forced vital capacity (FVC) by 67.4%. Although proteinuria was observed, no renal dysfunction or hypertension was present.

**Figure 2:**
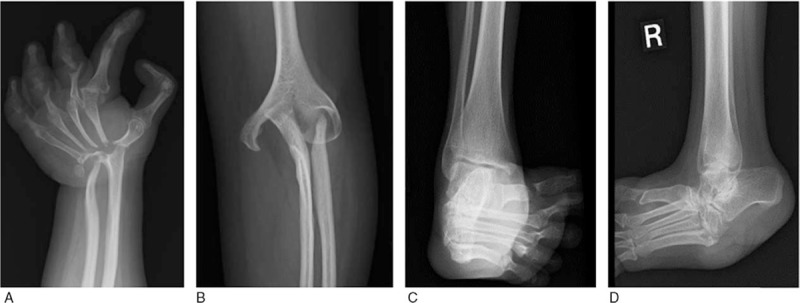
X-ray images of (A) hand joints; (B) elbow joint; (C) ankle joint; (D) foot.

X-ray images of the limbs showed remarkable osteolysis in joints of the hand, elbow, and ankle (Fig. 2).

**Figure 3:**
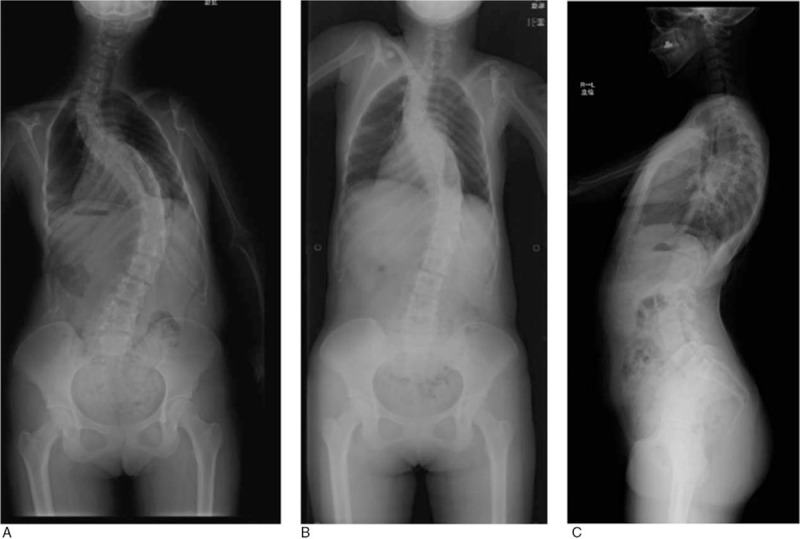
X-ray images. (A) Standing position (P→A); (B) recumbent-traction position (P→A); (C) standing position (sagittal).

The major curve of scoliosis was 82° (MC, Th7-L2; Th11 apical vertebra), and the upper curve was 77° ay (UC, Th1-6; Th3 apical vertebra). In a recumbent-traction position, major curve was 54° and upper curve was 56°. The results of sagittal plane measurement were as follows. Sagittal vertical axis: 30 mm; pelvic tilt (PT), 17°; sacral slope (SS), 60°; lumbar lordosis (LL), 71°; and pelvic incidence (PI), 77°. In terms of sagittal plane alignment, the PI–LL angle was <10° and no mismatch was observed; thoracic kyphosis was decreased to 16° (Fig. 3).

**Figure 4:**
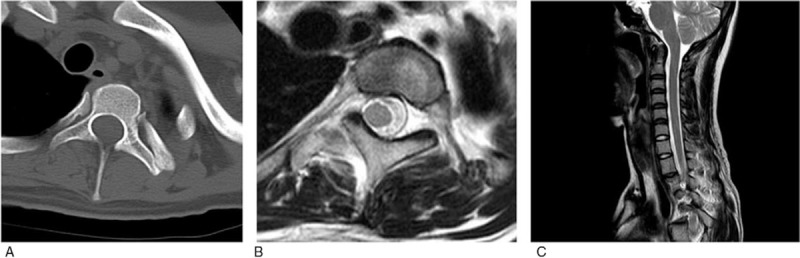
(A) CT axial Th2 spine level; (B) MRI T2WI axial Th2 spine level; (C) MRI T2WI sagittal. CT = computerized tomography, MRI = magnetic resonance imaging.

Computerized tomography revealed a partially narrowed vertebral arch, with no findings suggesting osteolysis. Magnetic resonance images showed deviation of the spinal cord, while neither Chiari malformation nor spina bifida was observed (Fig. 4).

## 3 Diagnostic assessment and therapeutic interventions

The patient was diagnosed with symptomatic scoliosis secondary to MCTO. The 2 rigid curves could not be corrected with traction or lateral bending. Moreover, due to progressive scoliosis and exacerbation in respiratory function, we decided to perform surgical treatment for the scoliosis.

After careful consideration based on computerized tomography (CT) imaging, we decided to perform correction and fusion from Th2 to L3 using a MESA system (K2M Inc., Leesburg, VA). For upper thoracic vertebrae with narrow pedicles, we used hooks to each pedicle for anchoring. For the rest of the vertebrae with pedicles of sufficient thickness, we inserted pedicle screws into each pedicle for anchoring. After performing Ponte osteotomy at each facet joint, we performed translation and in situ bending as spinal corrections. There were no abnormal findings in intraoperative spinal cord monitoring

**Figure 5:**
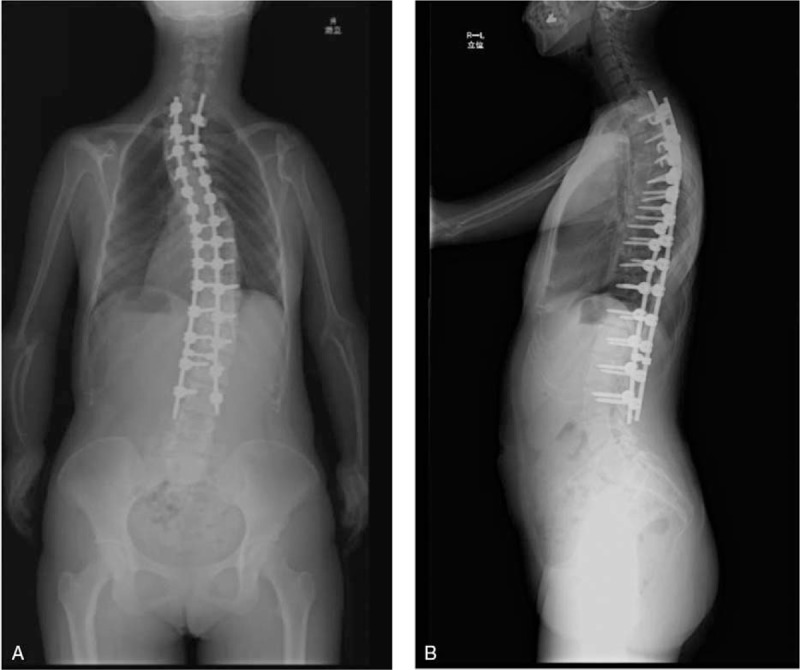
(A) Standing position (front); (B) standing position (side).

Postoperative x-ray demonstrated scoliosis angle correction from 77° to 38° at the upper curve and 82° to 39° at the major curve. Postoperative x-ray demonstrated thoracic kyphosis angle correction from 16° to 21°. The patient's height increased from 155 to 161 cm (Fig. 5).

We did not perform external fixation after the surgical procedure and the patient started ambulation the following day. The patient started to walk independently after 2 weeks and was discharged from the hospital after 4 weeks. Because her upper limbs had shortened, she required some devices to pull up her pants. It has been 12 months since the operation and no exacerbation has been observed.

## 4 Discussion

MCTO is a rare hereditary disease caused due to mutations in the *MafB* gene, a negative regulator of osteoclastogenesis. Kohler et al^[8]^ reported MCTO cases where patients were treated with external fixation and without surgical treatment. To the best of our knowledge, there have not been any reported cases in which the patient received surgical treatment.

The patient may have suffered from adolescent idiopathic scoliosis (AIS) secondary to MCTO. However, the upper positions of the curves were not typical for AIS. Computed tomography revealed a lytic change in the costovertebral and zygapophyseal joints. Thus, we diagnosed her with MCTO complicated with symptomatic scoliosis. We concluded that the patient needed surgery because aggravation of the symptoms was expected.

Yamaguchi et al^[9]^ have pointed out 3 advantages of 2-stage anterior release and posterior fusion: a higher correction rate, fewer complications (such as spinal cord injury), and lesser pseudarthrosis at a fixed point. According to Byrd et al,^[10]^ to achieve maximal scoliosis correction in adult patients with a rigid curve, a posterior-only approach is insufficient, and a two-stage anterior–posterior approach is required. However, as reported by Crostelli et al^[11]^ and Pourfeizi et al,^[12]^ a posterior-only approach achieved similar correction compared with a combined anterior–posterior approach while avoiding the risk of respiratory function disorder or operative stress caused in an anterior approach.

In the present case, the patient was suffering from lung and kidney disorders. Therefore, operative stress had to be avoided. Because the Cobb angle of her lateral spinal curvature was approximately 80°, a drastic correction was not required. Based on the above reasons, we selected a posterior-only approach.

We accomplished adequate correction of scoliosis. On the other hand, because the curve of her spine could be corrected regardless of the shortness of her limbs, her arm's reach drastically changed. Consequently, the outcome of the treatment resulted in an inconvenient lifestyle (e.g., dependence on a device to wear pants). Considering the possibility of such situations, not only the effectiveness of the treatment but also the QOL of treated patients should be closely monitored. To the best of our knowledge, there have been no other reports on surgical treatment of scoliosis secondary to MCTO using a posterior-only approach. During the operation, fixation of the pedicle screws was favorable. Moreover, although still within short-term follow-up, her clinical course has been benign and the alignment has also been favorable. In this context, based on our bibliographic survey, there are no other reports of synostosis subsequent to an operation to treat MCTO. Therefore, further follow-up is thought to be necessary.

## 5 Conclusion

We performed successful surgical treatment of syndromic scoliosis secondary to MCTO. To our knowledge, this is the first report on such a treatment. Our data suggest that a posterior-only approach can achieve favorable correction.

## 6 Ethical review

Ethical approval is unnecessary because the patient and her family have signed the informed consent before some important diagnosis and treatment. We took written informed consent for this publication.

## Acknowledgments

This case report was prepared according to the CARE guidelines.

## Author contributions

**Conceptualization:** Kunihiko Miyazaki, Satoshi Komatsubara.

**Data curation:** Kunihiko Miyazaki.

**Formal analysis:** Kunihiko Miyazaki, Satoshi Komatsubara.

**Investigation:** Kunihiko Miyazaki, Satoshi Komatsubara.

**Methodology:** Kunihiko Miyazaki, Satoshi Komatsubara.

**Project administration:** Satoshi Komatsubara.

**Resources:** Kunihiko Miyazaki, Satoshi Komatsubara, Koki Uno, Tetsuji Yamamoto.

**Supervision:** Tetsuji Yamamoto.

**Writing – original draft:** Kunihiko Miyazaki.

**Writing – review & editing:** Satoshi Komatsubara, Koki Uno, Ryuji Fujihara, Tetsuji Yamamoto.
